# Beef Intake Is Associated with Higher Nutrient Intake and Nutrient Adequacy in U.S. Adolescents, NHANES 2001–2018

**DOI:** 10.3390/nu15234996

**Published:** 2023-12-02

**Authors:** Kristin Fulgoni, Victor L. Fulgoni

**Affiliations:** Nutrition Impact, LLC, Battle Creek, MI 49014, USA; fulgonik@gmail.com

**Keywords:** adolescents, beef, nutrient adequacy, NHANES

## Abstract

Nutrient adequacy among adolescents is of concern due to higher nutrient requirements for their developing bodies as well as the gap between the current nutrient intake and the recommendations. The objective of this study was to determine beef intake and assess the relationship between beef consumption and nutrient intake and nutrient adequacy in male and female adolescents, 14–18 years of age. Dietary recalls collected during the What We Eat in America (WWEIA) portion of the National Health and Nutrition Examination Survey (NHANES) cycles 2001–2018 were utilized to determine beef intake. Usual nutrient intakes were determined with the National Cancer Institute method in conjunction with day 1 and day 2 total nutrient files. Nutrient adequacy was assessed by calculating the percentage of the population below the estimated average requirement (EAR) or above the adequate intake (AI). The average beef intake of male and female adolescent beef consumers was 57.9 ± 2.4 and 46.8 ± 2.2 g with a 90th percentile of 82.3 ± 4.3 and 67.8 ± 3.5 g, respectively. Compared to non-consumers, beef consumers had a 10% or higher intake of calcium, iron, phosphorus, potassium, total choline, vitamin B12, and zinc. Over 50% of the adolescent population (regardless of beef consumption) had intakes below the EAR for calcium, magnesium, and vitamins A, C, D, and E. The percentage of the beef-consuming population below the EAR was lower for calcium, copper, folate, iron, phosphorus, zinc, and vitamins B12 and B6 as compared to non-consumers. Additionally, the portion of the population above the AI for sodium was higher in female beef consumers as compared to non-consumers. We estimate approximately 900,000 to 1,400,000, 400,000–700,000, 200,000–600,000, and 200,000–400,000 fewer adolescents to be below the EAR for zinc, phosphorus, vitamin B12, and iron, respectively if beef non-consumers were to consume beef. This study suggests beef can help increase the nutrient intake and nutrient adequacy in the diets of adolescents, helping to close important gaps for this nutritionally vulnerable population. While recommendations to reduce beef intake are widely prevalent, this could result in unintended nutritional consequences regarding under-consumed nutrients including those of public health concern important for adolescent health.

## 1. Introduction

Nutrient inadequacy in adolescents is of great concern due to higher nutrient needs for their developing bodies including those needed for pubertal maturation, sexual development, and rapid growth [1]. During this period of rapid growth, bone and muscle mass as well as height and body weight increase, resulting in a larger blood volume, therefore requiring higher levels of protein, iron, zinc, and B vitamins [1,2]. Pubertal maturation and menstruation in females also increase the requirement of iron due to blood loss. Despite this knowledge, iron deficiency and iron-deficiency-related anemia remain relatively common in female adolescents, with iron-deficiency-related anemia being the leading cause of disability-adjusted life years lost in this population [1,3]. Other vital nutrients are being underconsumed by the adolescent population. According to the Dietary Guidelines for Americans Advisory Committee (DGAC), both male and female adolescents consume less than the recommended intake for phosphorus, magnesium, and choline, with females also having low intake of protein, iron, folate, and vitamins B6 and B12 [4]. In addition, adolescents have the lowest percentage of the population taking dietary supplements, which reiterates the need for increased consumption of nutrient-dense foods [4].

In addition to nutrient inadequacy, diet quality is also less than desired in adolescents, especially females. The 2020–2025 Dietary Guidelines for Americans reported that not only do adolescents have lower diet quality than their younger counterparts, they also have the greatest disparities between the recommended and current nutrient intakes of all children, as such they are at greater risk of nutrient inadequacy [5]. On the other hand, the HEI-2010 total protein foods component score was higher for those 14–18 y compared to younger children but was still 5% below the maximum score suggesting further protein consumption could help boost overall diet quality [6]. The decrease in diet quality between young children and adolescents is likely impacted by the increased time outside of the house leading to more independence in choosing their own diet [5]. Additionally, adolescents commonly adjust their dietary patterns in response to various factors such as peer influences, social media, and body image issues which can lead to meal skipping and restrictive eating, especially in females [7,8,9]. Furthermore, a recent report has identified the undernutrition of adolescent girls (and women of child-bearing age) as a global problem, and progress to improve this situation has been too slow and may be further hampered by geopolitical issues [10].

Beef is a nutrient-dense protein source that provides several nutrients critical to the American diet with approximately 50% of the total population being consumers and 67% of adolescents 12–19 y consuming beef in 2009–2010 [11,12]. Two previous studies focused on adolescents and adults, separately, reported beef provided 14–16% of daily protein, approximately 20–25% of vitamin B12 and zinc, as well as more than 5% of total fat, saturated fat, choline (adults only), niacin, vitamin B6, iron, phosphorus, and potassium [13,14]. Discrepancies in beef and protein intake between sexes is prevalent in the adolescent life stage with beef and total meat intake being lower in female adolescents and adults, as compared to their male counterparts [5,14]. This likely contributes to a lower protein intake overall as well as higher nutrient inadequacy, with 23% of adolescent females being below the estimated average requirement (EAR) for protein as compared to 8% of male adolescents, as recently reported by the USDA [15]. In addition to differences in protein and beef intake, male adolescents (12–19 y) consume more fruit, grains, and dairy as compared to females, with intakes of grains and dairy being 29 and 55% higher, respectively [16]. These differences in dietary patterns further contribute to nutrient adequacy discrepancies between sexes, with double the percentage of the female adolescent population falling below nutrient recommendations for several nutrients including thiamin, riboflavin, vitamin B6, zinc, and copper as compared to males [15]. Including beef in the adolescent diet could help alleviate protein and iron inadequacy while also contributing considerably to several other micronutrients with inadequate intake. Because nutrient requirements are higher in this age group, and large gaps are present between current and recommended dietary intakes, the aim of this study was to update a previous report on adolescent beef and nutrient intake [13] but also to examine nutrient adequacy measured as percentage of the population below the EAR of adolescent beef consumers and non-consumers using data from NHANES 2001–2018.

## 2. Materials and Methods

### 2.1. Data Source and Study Population

The National Health and Nutrition Examination Survey (NHANES) is a government survey focused on assessing the health and nutritional status of the non-institutionalized United States population. A core segment of NHANES is the dietary interview portion, What We Eat in America (WWEIA), which consists of two 24 h dietary recalls administered by trained staff. WWEIA utilizes the automated multiple pass method, which increases the accuracy of dietary reporting [17]. All participants complete the first 24 h dietary recall, whereas most, but not all, are asked to participate in the second 24 h dietary interview via the phone. For this study, beef consumers were defined as subjects who reported beef intake on either the day 1 or day 2 recalls. Details on the NHANES’ methods and procedures are available on their website [18,19].

The current study included male and female adolescents, 14–18 years of age (y), who participated in NHANES cycles 2001–2018 (the latest full sets of data available). Those with incomplete or unreliable dietary recalls as well as those pregnant and/or lactating were removed from the sample, resulting in a final sample size of 2561, which is representative of the American adolescent population of over 21.1 million. This age group was focused on due to their increased autonomy over their diets and higher nutrient requirements to support physical growth.

### 2.2. Determination of Beef Intake

Beef content of foods reported in WWEIA was determined by applying previously utilized methods [14]. Briefly, the Food Patterns Equivalents Database (FPED) and Food Patterns Ingredient Database (FPID) were combined with the Food and Nutrient Database for Dietary Studies (FNDDS) food and ingredient codes to quantify beef content of foods. For FPID variables that contain beef (protein foods—meats, protein foods—cured meat), the descriptions were examined to determine the percentage of beef within each ingredient. If the ingredient containing beef was determined to be entirely beef (e.g., ground beef), the value was multiplied by 100%. For ingredients that contain a second or third type of meat indicated by the description (e.g., bologna, beef and pork) a multiplication factor was employed to capture the beef portion.

The ingredient profile of several FNDDS food codes contained ingredients with missing FPID values or the food was a modified food (e.g., low-sodium, lower fat, etc.). For food codes with missing FPID values, the ingredient profile was replaced with complete profiles of the same food code from another NHANES cycle or a replacement ingredient code was utilized by identifying another ingredient code with a similar description.

### 2.3. Nutrient Intake Calculations

Nutrient intakes were obtained from the day 1 and day 2 total nutrient files. The USDA provides the nutrient composition of all foods reported as consumed and factors in processing techniques as well as the addition of items in preparation (fats, salt, sugar, etc.). The National Cancer Institute (NCI) method was used to estimate the usual intakes and the distribution of those intakes in beef consumers and non-consumers. Both 24 h recalls were utilized to determine the usual intake of beef using the two-part model (frequency and amount) [20]. The nutrient usual intake calculations used both 24 h recalls and the one-part (amount) method since most subjects consume some amount of all nutrients daily [21]. The cut-point method was utilized to determine the percentage of the population below the EAR and above the adequate intake (AI). Although dietary supplements can dramatically impact nutrient intake and possibly affect the efficacy of nutrient absorption, they were not included in the analyses as we were interested in intake from foods only, and only about 35% of children 1–18 y report use of dietary supplements [22].

### 2.4. Statistical Analyses

All analyses were adjusted for NHANES complex sample design and performed with SAS 9.4 (SAS Institute, Cary, NC, USA). Regression analyses were utilized to determine the differences between the nutrient intake of beef consumers and non-consumers using PROC SURVEYREG of SAS. Covariates age, sex, ethnicity, and energy were utilized for nutrient intake analyses. Differences in the percentage of the population below EAR/above AI were determined via *t*-tests. *p* < 0.01 was used for significance.

## 3. Results

### 3.1. Beef Intake

Female adolescent beef consumers had a mean intake of 46.8 ± 2.21 g per day (g/d) of beef (Table 1). Their median intake was 44.8 ± 2.29 g/d and had a 90th percentile intake of 67.8 ± 3.46 g/d. On the other hand, male adolescent beef consumers had approximately 24% higher beef intake than females at 57.9 ± 2.67 g/d. Male adolescent beef consumers had a median and 90th percentile intake of 55.8 ± 2.72 and 82.3 ± 4.29 g/d, respectively. Demographics for the study population are available in Supplementary Table S1.

### 3.2. Nutrient Intake

Female adolescent beef consumers had higher intakes of calcium (159 ± 34.5 mg), folate (DFE; 63.1 ± 21.7 µg), iron (1.99 ± 0.54 mg), niacin (2.64 ± 0.84 mg), phosphorus (174 ± 48.4 mg), potassium (216 ± 70.8 mg), riboflavin (0.20 ± 0.07 mg), selenium (16.2 ± 3.44 µg), sodium (574 ± 97.1 mg), thiamin (0.18 ± 0.06 mg), total choline (36.7 ± 9.71 mg), vitamin B12 (1.25 ± 0.22 µg), and zinc (2.99 ± 0.36 mg) as compared to non-consumers (Table 2).

Similarly, male adolescent beef consumers had higher intakes of calcium (213 ± 42.2 mg), iron (2.48 ± 0.83 mg), niacin (3.23 ± 1.20 mg), phosphorus (241 ± 54.4 mg), potassium (313 ± 90.7 mg), selenium (23.3 ± 3.93 µg), sodium (821 ± 115 mg), thiamin (0.24 ± 0.08 mg), total choline (46.1 ± 12.7 mg), vitamin B12 (1.46 ± 0.33 µg), and zinc (3.88 ± 0.51 mg) as compared to non-consumers (Table 3). In contrast to female adolescents, no differences in intake of folate and riboflavin were observed between male adolescent beef consumers and non-consumers.

### 3.3. Nutrient Adequacy

Over 50% of the adolescent population (regardless of beef consumption) had total dietary intakes below the EAR for calcium, magnesium, and vitamins A, C, D, and E (Table 4).

Additionally, female adolescent beef consumers had a lower percentage of the population below the EAR for calcium (difference between consumers and non-consumers: 13.4 ± 3.68%), copper (16.0 ± 3.70%), folate (DFE; 16.7 ± 4.53%), iron (14.0 ± 2.71%), niacin (4.37 ± 1.45%), phosphorus (24.7 ± 6.01%), riboflavin (8.01 ± 2.15%), thiamin (11.1 ± 2.69%), vitamin B12 (20.4 ± 3.12%), vitamin B6 (10.9 ± 3.02%), and zinc (46.2 ± 5.12%) (Table 4). For sodium, a higher percentage of the consumer population was above the AI (4.16 ± 1.30%) as compared to non-consumers.

As for male adolescents, beef consumers had a lower percentage of the population below the EAR for calcium (23.9 ± 4.95%), copper (13.3 ± 3.91%), folate (DFE; 12.7 ± 4.27%), iron (9.02 ± 2.66%), phosphorus (25.0 ± 5.07%), riboflavin (8.48 ± 2.25%), thiamin (8.69 ± 3.03%), vitamins B12 (11.2 ± 2.58%) and B6 (8.96 ± 2.81%), and zinc (50.7 ± 6.60%) as compared to non-consumers (Table 5). Contrary to the results observed in female adolescents, no differences in the percentage of the population below the EAR/above the AI were present between male adolescent beef consumers and non-consumers for niacin and sodium.

## 4. Discussion

The current study showed there is a considerable percentage of adolescents 14–18 y (consumers and non-consumers) not meeting nutrient recommendations, with greater than 50% of the total population below the EAR for calcium, magnesium, and vitamins A, C, D, and E and less than 30% of the total population above the AI for potassium and total choline. On the other hand, adolescent beef consumers had higher intakes of several critical nutrients including vitamin B12 and zinc, as well as iron, calcium, and potassium, which are nutrients of public health concern. As a result, beef consumers had a higher percentage of the population meeting the nutrient recommendations for iron, folate, riboflavin, thiamin, vitamins B12 and B6, zinc, calcium, copper, and phosphorus than non-consumers. Additionally, beef consumers had higher intakes of sodium as well as a higher percentage of the population above the AI for sodium (in females only). This study suggests beef can contribute significantly to both the nutrient intake and nutrient adequacy of several critical nutrients in adolescents of this age, especially females who are particularly vulnerable to nutrient inadequacy.

To further assess the possible impact of beef consumption on a population basis, we estimated the number of adolescent non-consumers that would no longer be below the EAR for several of the underconsumed nutrients, namely zinc, phosphorus, vitamin B12, and iron, if they incorporated beef into their diet and consumed a food pattern similar to beef consumers. We multiplied the difference in consumer and non-consumers for nutrient adequacy for these nutrients (see Table 4 and Table 5) by the population of non-consumers (3.0 million females and 1.8 million males). Assuming non-consumers would consume beef and follow a similar dietary pattern as current beef consumers, we would expect approximately 1,400,000, 730,000, 600,000, and 420,000 fewer female adolescents and 890,000, 440,000, 200,000, and 160,000 fewer male adolescents to be below the EAR for zinc, phosphorus, vitamin B12, and iron, respectively.

While replacing or reducing beef intake has been a common topic within the nutrition community due to possible benefits to health and the environment [23], reducing beef intake may have unintended nutritional consequences, particularly regarding underconsumed nutrients and nutrients of public concern for adolescent health. The current study found beef consumers had upwards of 15% more intake of calcium, iron, phosphorus, selenium, sodium, total choline, vitamin B12, and zinc than non-consumers, which is in agreement with several previous studies. Studies focusing on adolescents using NHANES 2011–2014, found meat provided high levels of iron, magnesium, and potassium, contributing between 2 and 3.6% of their daily intake, as well as 2.6–4% of daily energy, sodium, and saturated fats [24,25]. On the other hand, modeling the impact of replacing 50% of meat with plant protein and increasing plant-based meat alternatives by 100% resulted in a decrease in protein, cholesterol, zinc, vitamin B12 while increasing fiber, polyunsaturated fatty acids, magnesium, and folate [26]. A second modeling study focused solely on female adolescents found increasing plant foods by 100% resulted in increased nutrient inadequacy for calcium, zinc, and protein, where the percentage of the population below the EAR nearly tripled [27]. Since adolescents have the largest difference between recommendations and current intakes as compared to all other age groups [5], encouraging adolescents, especially females, to consume beef could help to narrow these gaps and promote healthy growth. Alternatively, in beef non-consumers other ways to obtain missing nutrients provided by beef (e.g., iron, phosphorus, choline, protein, zinc, vitamin B12) will need to be identified.

We were unable to find any other studies examining nutrient adequacy (percentage below the EAR/above the AI) in adolescents based on beef consumption. One study did report differences in nutrient adequacy in Canada based on red meat consumption showing that those that did not consume red meat were at higher risk for inadequate intakes for energy, calcium, vitamin D, and potassium, while those that consumed red meat were at higher risk of inadequacy for dietary fiber, vitamin A, and magnesium [28]. Unfortunately, this group did not separate results by age groups but did indicate there may be different results for those with higher nutrient needs like children. One other study recently reported pork consumers had a higher percentage of the population meeting the recommended levels of calcium, choline, copper, iron, phosphorus, potassium, riboflavin, thiamin, vitamin B12, and zinc as compared to non-consumers in children 2–18 y [29]. The results from these previous studies in conjunction with the current study’s findings suggest red meat, including beef, can increase nutrient adequacy in the U.S. population.

While the current study showed adolescent beef consumption was associated with a higher nutrient intake for beef specific nutrients as compared to non-consumers, such as iron, B vitamins, and zinc, it was also associated with higher nutrient intakes of other nutrients not necessarily in beef. The current study found beef consumers had a higher calcium intake as compared to beef non-consumers. This is likely due to the overall dietary pattern and possibly differences in the intake of recommended food groups. Regardless of beef being a part of adolescent diets, there is still considerable opportunity for improvement of nutrient intake to decrease nutrient inadequacy in this population. Current research focusing on interventions and educational programs [30] in this age group are imperative to their health and wellbeing.

This study has several strengths including the use of several cycles of NHANES, a large nationally representative dataset. Additionally, the method for determining foods containing beef and therefore beef consumers has been previously published [14]. On the other hand, this study has several limitations. NHANES has a cross-sectional design, and therefore, causal relationships are unable to be assessed. The dietary recalls are based on memory and are known to be subject to misreporting [31]. While under-reporting has been associated with protein intake [32], the prevalence of misreporting in adolescents is not well understood making it difficult to determine the influence of reporting status on the study results. While NHANES and this study utilize two 24 h dietary recalls, it is possible participants consumed beef on days other than those reported, which would result in the current study underestimating beef intake. Additionally, the association of beef intake with higher nutrient intake and lower nutrient inadequacies may be due to beef but also may be due, at least in some part, to other foods that are consumed with beef. We also did not stratify our results by race/ethnicity, socioeconomic, or other demographic variables, which may influence the current results, as the contribution of beef to those in certain groups may differ.

Future research could include examining whether the results in the current study also apply to specific race/ethnicity groups, weight status groups, levels of education, various levels of socioeconomic status (household income), or those with certain lifestyle factors. Determining whether the results apply to different cultural backgrounds and religious beliefs would also be of merit since these factors typically influence dietary patterns [5]. Additionally, comparing the nutrient intake and nutrient adequacy of beef consumers to that of vegan/vegetarian adolescents would be of merit, since these dietary patterns are increasing in popularity, and more children are raised with these dietary patterns. Finally, it would be worthwhile to assess whether adolescent dietary supplement use addresses nutrient inadequacy from foods alone.

## 5. Conclusions

In conclusion, adolescent beef consumers had a higher nutrient intake as well as higher nutrient adequacy for numerous nutrients, including several nutrients of public health concern. Accordingly, it appears beef can help increase the nutrient intake and nutrient adequacy in diets of adolescents, especially that of females. Due to their rapid growth and increased nutrient requirements, care should be taken to ensure dietary recommendations that recommend less beef can replace nutrients that could be expected to be provided by beef (e.g., iron, phosphorus, choline, protein, zinc, vitamin B12) in adolescents.

## Figures and Tables

**Table 1:** Beef usual intake ^1^ by adolescent beef consumers (14–18 y).

Beef Intake	Females (N = 852)		Males (N = 1015)	
	g/d	oz/d	g/d	oz/d
Mean	46.81 (2.21)	1.65 (0.08)	57.91 (2.67)	2.04 (0.09)
Median	44.80 (2.29)	1.58 (0.08)	55.79 (2.72)	1.97 (0.10)
90th Percentile	67.78 (3.46)	2.39 (0.12)	82.27 (4.29)	2.90 (0.15)

^1^ Data Source: NHANES 2001–2018. Data presented as mean (SE). g/d: gram per day; oz/d: ounce per day.

**Table 2:** Usual intake ^1^ of nutrients by beef consumers ^2^ compared to non-consumers in female adolescents (14–18 y).

Nutrients	Consumer (N = 852)	Non-Consumer (N = 441)	Consumer vs. Non-Consumer	
	Mean (SE)	Mean (SE)	Beta (SE)	*p*
Calcium (mg)	945.3 (20.9)	785.9 (27.4)	159.4 (34.5)	**<0.0001**
Copper (mg)	0.95 (0.02)	0.88 (0.03)	0.07 (0.03)	0.0372
Folate, DFE (µg)	492.2 (11.1)	429.1 (18.7)	63.12 (21.7)	**0.0036**
Iron (mg)	13.79 (0.25)	11.80 (0.48)	1.99 (0.54)	**0.0003**
Magnesium (mg)	233.0 (3.90)	220.2 (7.25)	12.75 (8.23)	0.1213
Niacin (mg)	22.67 (0.54)	20.04 (0.65)	2.64 (0.84)	**0.0017**
Phosphorus (mg)	1239 (25.4)	1064 (41.2)	174.3 (48.4)	**0.0003**
Potassium (mg)	2084 (36.4)	1868 (60.7)	215.8 (70.8)	**0.0023**
Riboflavin (Vitamin B2) (mg)	1.80 (0.04)	1.60 (0.06)	0.20 (0.07)	**0.0032**
Selenium (µg)	100.4 (2.05)	84.19 (2.76)	16.21 (3.44)	**<0.0001**
Sodium (mg)	3202 (61.3)	2628 (75.3)	574.4 (97.1)	**<0.0001**
Thiamin (Vitamin B1) (mg)	1.51 (0.03)	1.33 (0.04)	0.18 (0.06)	**0.0010**
Total choline (mg)	250.0 (5.27)	213.3 (8.16)	36.70 (9.71)	**0.0002**
Vitamin A (RE)	505.4 (14.0)	511.8 (24.2)	−6.49 (28.0)	0.8167
Vitamin B12 (µg)	4.59 (0.13)	3.34 (0.17)	1.25 (0.22)	**<0.0001**
Vitamin B6 (mg)	1.71 (0.05)	1.60 (0.07)	0.11 (0.08)	0.1742
Vitamin C (mg)	61.78 (2.84)	60.37 (4.03)	1.41 (4.93)	0.7753
Vitamin D (D2 + D3) (µg)	4.01 (0.14)	3.80 (0.22)	0.21 (0.26)	0.4301
Vitamin E (ATE) (mg)	7.17 (0.16)	6.96 (0.35)	0.21 (0.39)	0.5891
Zinc (mg)	10.18 (0.20)	7.18 (0.31)	2.99 (0.36)	**<0.0001**

^1^ Data Source: NHANES 2001–2018. ^2^ Beef consumers were defined as subjects with beef intake on day 1 or day 2.

**Table 3:** Usual intake ^1^ of nutrients by beef consumers ^2^ compared to non-consumers in male adolescents (14–18 y).

Nutrients	Consumer (N = 1015)	Non-Consumer (N = 253)	Consumer vs. Non-Consumer	
	Mean (SE)	Mean (SE)	Difference (SE)	Difference (SE)
Calcium (mg)	1098 (23.6)	885.1 (35.0)	213.1 (42.2)	**<0.0001**
Copper (mg)	1.06 (0.02)	0.97 (0.05)	0.10 (0.05)	0.0588
Folate, DFE (µg)	565.9 (14.8)	489.4 (27.3)	76.51 (31.1)	0.0138
Iron (mg)	16.12 (0.29)	13.63 (0.78)	2.48 (0.83)	**0.0029**
Magnesium (mg)	263.0 (4.21)	240.3 (11.7)	22.74 (12.4)	0.0674
Niacin (mg)	27.03 (0.56)	23.80 (1.06)	3.23 (1.20)	**0.0073**
Phosphorus (mg)	1452 (25.2)	1211 (48.2)	240.8 (54.4)	**<0.0001**
Potassium (mg)	2388 (37.7)	2075 (82.5)	313.5 (90.7)	**0.0005**
Riboflavin (Vitamin B2) (mg)	2.11 (0.05)	1.87 (0.09)	0.24 (0.10)	0.0245
Selenium (µg)	118.3 (2.10)	95.04 (3.32)	23.25 (3.93)	**<0.0001**
Sodium (mg)	3755 (66.6)	2934 (93.4)	821.4 (115)	**<0.0001**
Thiamin (Vitamin B1) (mg)	1.78 (0.04)	1.54 (0.07)	0.24 (0.08)	**0.0025**
Total choline (mg)	291.7 (5.95)	245.6 (11.2)	46.05 (12.7)	**0.0003**
Vitamin A (RE)	574.1 (14.9)	563.1 (26.5)	11.01 (30.4)	0.7172
Vitamin B12 (µg)	5.58 (0.16)	4.12 (0.28)	1.46 (0.33)	**<0.0001**
Vitamin B6 (mg)	2.03 (0.05)	1.89 (0.11)	0.13 (0.12)	0.2520
Vitamin C (mg)	65.16 (2.73)	65.11 (4.86)	0.05 (5.57)	0.9932
Vitamin D (D2 + D3) (µg)	4.90 (0.13)	4.58 (0.33)	0.32 (0.36)	0.3753
Vitamin E (ATE) (mg)	7.92 (0.16)	7.91 (0.56)	0.01 (0.58)	0.9854
Zinc (mg)	12.03 (0.24)	8.14 (0.45)	3.88 (0.51)	**<0.0001**

^1^ Data Source: NHANES 2001–2018. ^2^ Beef consumers were defined as subjects with beef intake on day 1 or day 2.

**Table 4:** Percentage of the population below the EAR or above the AI in beef consumers ^1^ compared to non-consumers in female adolescents (14–18 y) ^2^.

Nutrients	Total Population (N = 1293)	Consumer (N = 852)	Non-Consumer (N = 441)	Consumer vs. Non-Consumer	
	Mean (SE)	Mean (SE)	Mean (SE)	Beta (SE)	*p*
Percent Below EAR ^3^					
Calcium (mg)	77.36 (2.10)	73.34 (2.73)	86.73 (2.46)	−13.39 (3.68)	**0.0003**
Copper (mg)	16.87 (1.74)	11.80 (1.51)	27.80 (3.38)	−16.00 (3.70)	**<0.0001**
Folate, DFE (µg)	17.38 (1.99)	11.88 (2.00)	28.57 (4.07)	−16.69 (4.53)	**0.0002**
Iron (mg)	12.98 (1.03)	8.63 (0.88)	22.63 (2.57)	−14.00 (2.71)	**<0.0001**
Magnesium (mg)	88.41 (1.62)	88.46 (1.61)	87.97 (3.55)	0.49 (3.90)	0.8997
Niacin (mg)	1.85 (0.48)	0.33 (0.21)	4.70 (1.43)	−4.37 (1.45)	**0.0026**
Phosphorus (mg)	36.48 (2.89)	28.65 (2.92)	53.36 (5.25)	−24.70 (6.01)	**<0.0001**
Riboflavin (Vitamin B2) (mg)	3.95 (0.82)	1.57 (0.49)	9.59 (2.10)	−8.01 (2.15)	**0.0002**
Selenium (µg)	0.73 (0.34)	0.11 (0.07)	2.03 (1.02)	−1.92 (1.02)	0.0606
Thiamin (Vitamin B1) (mg)	6.62 (1.06)	3.23 (0.92)	14.33 (2.53)	−11.10 (2.69)	**<0.0001**
Vitamin A (RE)	50.46 (2.46)	50.34 (3.01)	50.70 (4.95)	−0.37 (5.80)	0.9497
Vitamin B12 (µg)	8.60 (1.15)	1.77 (0.61)	22.20 (3.06)	−20.43 (3.12)	**<0.0001**
Vitamin B6 (mg)	7.78 (1.22)	4.41 (1.14)	15.31 (2.80)	−10.90 (3.02)	**0.0003**
Vitamin C (mg)	50.86 (3.11)	50.05 (3.97)	51.97 (4.95)	−1.92 (6.35)	0.7619
Vitamin D (D2 + D3) (µg)	98.67 (0.26)	98.92 (0.31)	98.20 (0.65)	0.72 (0.72)	0.3198
Vitamin E (ATE) (mg)	96.40 (0.96)	97.53 (0.76)	94.29 (2.52)	3.24 (2.63)	0.2174
Zinc (mg)	26.37 (2.42)	11.22 (1.89)	57.46 (4.76)	−46.24 (5.12)	**<0.0001**
Percent Above AI ^3^					
Potassium (mg)	27.30 (2.38)	30.49 (2.53)	20.53 (3.60)	9.96 (4.40)	0.0237
Sodium (mg)	98.54 (0.49)	99.92 (0.05)	95.75 (1.30)	4.16 (1.30)	**0.0014**
Total choline (mg)	2.08 (0.50)	2.13 (0.61)	1.77 (0.87)	0.36 (1.06)	0.7314

^1^ Beef consumers were defined as subjects with beef intake on day 1 or day 2. ^2^ Data Source: NHANES 2001–2018. ^3^ EAR: estimated average requirement; AI: adequate intake.

**Table 5:** Percentage of the population below the EAR or above the AI in beef consumers ^1^ compared to non-consumers in male adolescents (14–18 y) ^2^.

Nutrients	Total Population (N = 1268)	Consumer (N = 1015)	Non-Consumer (N = 253)	Consumer vs. Non-Consumer	
	Mean (SE)	Mean (SE)	Mean (SE)	Difference (SE)	Difference (SE)
Percent Below EAR ^3^					
Calcium (mg)	58.90 (2.62)	53.92 (3.07)	77.78 (3.89)	−23.86 (4.95)	**<0.0001**
Copper (mg)	8.03 (1.06)	5.08 (0.99)	18.38 (3.78)	−13.29 (3.91)	**0.0007**
Folate, DFE (µg)	7.20 (1.28)	4.83 (1.18)	17.57 (4.11)	−12.74 (4.27)	**0.0029**
Iron (mg)	2.66 (0.64)	1.15 (0.29)	10.17 (2.64)	−9.02 (2.66)	**0.0007**
Magnesium (mg)	89.52 (1.53)	89.43 (1.50)	91.29 (4.13)	−1.87 (4.40)	0.6709
Niacin (mg)	0.50 (0.19)	0.10 (0.07)	2.48 (0.97)	−2.38 (0.97)	0.0142
Phosphorus (mg)	15.85 (1.71)	10.90 (1.51)	35.90 (4.84)	−25.00 (5.07)	**<0.0001**
Riboflavin (Vitamin B2) (mg)	3.22 (0.58)	1.54 (0.40)	10.03 (2.21)	−8.48 (2.25)	**0.0002**
Selenium (µg)	0.14 (0.08)	0.01 (0.01)	0.66 (0.42)	−0.65 (0.42)	0.1230
Thiamin (Vitamin B1) (mg)	3.42 (0.71)	1.49 (0.45)	10.19 (3.00)	−8.69 (3.03)	**0.0041**
Vitamin A (RE)	64.63 (2.56)	65.21 (2.88)	66.03 (4.35)	−0.82 (5.22)	0.8753
Vitamin B12 (µg)	2.55 (0.56)	0.40 (0.16)	11.62 (2.57)	−11.23 (2.58)	**<0.0001**
Vitamin B6 (mg)	4.02 (0.72)	2.25 (0.62)	11.20 (2.74)	−8.96 (2.81)	**0.0014**
Vitamin C (mg)	55.17 (3.15)	54.69 (3.56)	55.45 (5.62)	−0.77 (6.65)	0.9083
Vitamin D (D2 + D3) (µg)	96.81 (0.56)	97.00 (0.61)	96.50 (1.31)	0.50 (1.45)	0.7276
Vitamin E (ATE) (mg)	93.83 (1.28)	94.69 (1.13)	89.80 (4.58)	4.89 (4.71)	0.2993
Zinc (mg)	19.50 (2.01)	9.47 (1.40)	60.16 (6.45)	−50.68 (6.60)	**<0.0001**
Percent Above AI ^3^					
Potassium (mg)	11.83 (1.50)	12.66 (1.63)	7.61 (2.51)	5.05 (2.99)	0.0915
Sodium (mg)	99.68 (0.15)	99.99 (0.01)	98.08 (0.80)	1.91 (0.80)	0.0168
Total choline (mg)	0.22 (0.11)	0.22 (0.13)	0.28 (0.25)	−0.06 (0.28)	0.8378

^1^ Beef consumers were defined as subjects with beef intake on day 1 or day 2. ^2^ Data Source: NHANES 2001–2018. ^3^ EAR: estimated average requirement; AI: adequate intake.

## Data Availability

The data presented in this study are from publicly available data in NHANES, and other additional data are available in the article.

## Supplementary Materials

The following supporting information can be downloaded at: https://www.mdpi.com/2072-6643/15/23/4996/s1, Table S1: Demographics ^1^ of beef consumers ^2^ compared to non-consumers in U.S. adolescents (14–18 y).
