# Supplementary material for: Beef Intake Is Associated with Higher Nutrient Intake and Nutrient Adequacy in U.S. Adolescents, NHANES 2001–2018
Source: Nutrients. 2023 Dec 2;15(23):4996. doi: 10.3390/nu15234996 (PMC10871076; doi:10.3390/nu15234996)
Supplement: Supplementary file 1 [file nutrients-15-04996-s001.zip › nutrients-2695618-supplementary.pdf]

Supplementary Table S1. Demographics[1] of beef consumers[2] compared to non-consumers in U.S. adolescents (14–18 y).

Demographic Variable	Total Population Mean (SE)	Non-Consumers Mean (SE)	Consumers Mean (SE)	Consumers vs. Non-consumers Beta (SE)	P
**Females (n=1,293)**					
Age (mean)	16.03 (0.06)	16.04 (0.12)	16.02 (0.08)	-0.02 (0.15)	0.8811
Ethnicity (%)					
Hispanic	22.35 (2.24)	23.08 (2.75)	21.99 (2.74)	-1.09 (3.40)	0.7498
Non-Hispanic White	52.36 (3.44)	47.35 (4.32)	54.80 (4.07)	7.44 (5.15)	0.1530
Non-Hispanic Black	15.38 (2.05)	15.11 (2.99)	15.51 (2.06)	0.39 (2.66)	0.8828
Non-Hispanic Asian	5.16 (0.92)	8.38 (1.64)	3.60 (0.94)	-4.77 (1.71)	**0.0069**
Poverty Income Ratio (PIR; %)					
PIR < 1.35	32.63 (2.87)	27.60 (3.61)	35.08 (3.56)	7.48 (4.57)	0.1068
1.35 ≤ PIR ≤ 1.85	11.31 (1.61)	10.13 (1.70)	11.89 (2.14)	1.76 (2.53)	0.4879
PIR > 1.85	56.06 (3.19)	62.27 (3.78)	53.03 (3.97)	-9.24 (4.78)	0.0576
Physical Activity (%)					
Sedentary	21.46 (1.88)	24.34 (2.91)	20.06 (2.60)	-4.28 (4.14)	0.3052
Moderate	32.32 (2.24)	28.41 (3.37)	34.21 (3.13)	5.80 (4.84)	0.2355
Vigorous	46.23 (2.47)	47.25 (3.54)	45.73 (2.97)	-1.52 (4.20)	0.7193
Smoking Status (%)					
Never	78.27 (2.55)	81.57 (3.53)	76.66 (2.90)	-4.91 (3.97)	0.2211
Current	2.37 (0.83)	2.12 (1.40)	2.49 (1.03)	0.37 (1.75)	0.8334
Weight Status (%)					
Overweight	16.32 (1.26)	18.40 (2.58)	15.32 (1.64)	-3.08 (3.30)	0.3543
Obese	21.74 (1.76)	20.02 (2.78)	22.57 (2.42)	2.55 (3.91)	0.5171
**Males (n=1,268)**					
Age (mean)	16.00 (0.06)	16.11 (0.15)	15.97 (0.07)	-0.14 (0.16)	0.3954
Ethnicity (%)					
Hispanic	24.84 (2.73)	25.35 (5.91)	24.71 (2.69)	-0.64 (5.76)	0.9121
Non-Hispanic White	49.91 (3.34)	45.07 (6.11)	51.11 (3.41)	6.05 (5.94)	0.3124
Non-Hispanic Black	14.56 (1.96)	16.46 (3.25)	14.10 (2.09)	-2.36 (3.32)	0.4800
Non-Hispanic Asian	4.76 (0.86)	9.54 (2.36)	3.58 (0.60)	-5.96 (2.06)	**0.0053**
Poverty Income Ratio (PIR; %)					
PIR < 1.35	33.06 (2.46)	29.14 (5.20)	33.95 (2.67)	4.80 (5.64)	0.3977
1.35 ≤ PIR ≤ 1.85	10.93 (1.56)	11.97 (3.54)	10.69 (1.61)	-1.28 (3.63)	0.7263
PIR > 1.85	56.01 (2.99)	58.89 (6.03)	55.36 (3.10)	-3.53 (6.11)	0.5657
Physical Activity (%)					
Sedentary	11.57 (1.36)	12.75 (3.59)	11.27 (1.51)	-1.47 (4.02)	0.7153
Moderate	26.77 (2.46)	33.62 (4.38)	25.08 (2.61)	-8.54 (4.60)	0.0683
Vigorous	61.66 (2.85)	53.63 (6.00)	63.65 (2.88)	10.01 (6.15)	0.1085
Smoking Status (%)					

Never	72.29 (2.24)	84.67 (3.52)	69.44 (2.55)	-15.23 (4.25)	**0.0007**
Current	4.86 (0.94)	0.94 (0.57)	5.81 (1.15)	4.87 (1.24)	**0.0002**
Weight Status (%)					
Overweight	15.76 (1.58)	13.55 (3.21)	16.31 (1.83)	2.76 (3.76)	0.4658
Obese	21.65 (1.80)	27.78 (4.97)	20.14 (1.85)	-7.64 (5.28)	0.1536

[1] Data Source: NHANES 2001-2018. [2] Beef consumers were defined as subjects with beef intake on day 1 or day 2.
